# A Soluble Receptor for Advanced Glycation End-Products Inhibits Hypoxia/Reoxygenation-Induced Apoptosis in Rat Cardiomyocytes via the Mitochondrial Pathway

**DOI:** 10.3390/ijms130911923

**Published:** 2012-09-20

**Authors:** Caixia Guo, Xiangjun Zeng, Juanjuan Song, Min Zhang, Hongxia Wang, Xiaowei Xu, Fenghe Du, Buxing Chen

**Affiliations:** 1 Department of Cardiology, Beijing Tian Tan Hospital, Capital Medical University, Beijing 100050, China; E-Mails: songjuan2008@163.com (J.S.); dayanjingzm@sina.com (M.Z.); xuxiaoweittyy@sina.com (X.X.); fhduu@yahoo.com.cn (F.D.); chbux@126.com (B.C.); 2 Department of Pathophysiology, Capital Medical University, Beijing 100069, China; E-Mails: megan_zeng@163.com (X.Z.); whxdy@sina.com (H.W.)

**Keywords:** sRAGE, apoptosis, ischemia, reperfusion, mitochondria

## Abstract

Severe myocardial dysfunction and tissue damage resulting from ischemia/reperfusion (I/R) is a common clinical scenario in patients with certain types of heart diseases and therapies such as thrombolysis, percutaneous coronary intervention, coronary artery bypass grafting, and cardiac transplantation. The underlining mechanism of endogenous cardiac protection after I/R injury has been a focus of current research. Growing evidences suggests that soluble receptor for advanced glycation end-products (sRAGE) has a cardioprotective effect; however, its role in I/R injury remains unclear. We hypothesized that exogenous administration of sRAGE during hypoxia/reoxygenation (H/R) induces cardioprotection by inhibiting cardiomyocyte apoptosis via multiple signals, involving mitochondrial membrane potential (MMP), the mitochondrial permeability transition pore (mPTP), mitochondrial cytochrome c, caspase-3, Bcl-2 and Bax. Neonatal rat cardiomyocytes underwent hypoxia for 3-h followed by 2-h reoxygenation or were treated with sRAGE for 10 min before H/R. Compared with H/R alone, sRAGE pretreatment reduced H/R-induced cardiomyocyte apoptosis from 27.9% ± 5.9% to 9.4% ± 0.7% (*p* < 0.05). In addition, sRAGE treatment significantly inhibited H/R-induced mitochondrial depolarization and mPTP opening, reduced mitochondrial cytochrome c leakage, caspase-3 and caspase-9 activity, and decreased the ratio of Bax to Bcl-2. Therefore, we conclude that the exogenous administration of sRAGE during H/R is involved in cardioprotection by inhibiting apoptosis via the mitochondrial pathway, which, if further confirmed *in vivo*, may have important clinical implications during H/R.

## 1. Introduction

Ischemic heart disease is one of the main causes of death worldwide, and is considered a global disease burden with the World Health Organization expecting it to surpass infectious diseases and become the leading cause of mortality by 2020 [1]. Severe myocardial dysfunction and tissue damage resulting from ischemia/reperfusion (I/R) is a common clinical scenario in patients with certain types of heart disease and therapies such as thrombolysis, percutaneous coronary intervention, coronary artery bypass grafting, and cardiac transplantation [2]. The phenomenon that reperfusion causes damage in addition to that caused by the ischemic insult is referred to as an I/R injury [2,3]. Mechanisms underlying myocardial I/R injury span a broad range of fundamental biological changes, including metabolic, ionic, inflammatory, oxidant stress and apoptosis. Investigators have focused on developing an intervention to reduce cell injury after I/R [4,5]. Recent studies have suggested that lethal reperfusion injury is caused by mitochondrial KATP channel opening and reactive oxygen species signaling, which may lead to augmented mitochondrial calcium accumulation and activation of mitochondrial permeability transition pore (mPTP) opening [6,7]. mPTP opening occurs during reperfusion and appears to be a key event in the death of cardiomyocytes after a sequence of I/R. Nonspecific opening of the mPTP in the inner mitochondrial membrane results in the collapse of mitochondrial membrane potential (Δψ_m_), uncoupling of the respiratory chain and efflux of cytochrome c and other pro-apoptotic factors, which may lead to apoptosis or necrosis [8]. This is considered one of the major contributors to myocardial injury after I/R. Thus, inhibition of mPTP opening is a critical factor for cardioprotection [7,9]. Growing evidence suggests that the myocardium adapts to I/R by synthesizing and responding to various stress-induced growth factors and cytokines; the identification of these endogenous homeostatic mechanisms may open new avenues to limit I/R injury [10]. Therefore, investigators have concentrated on understanding the mechanism of the endogenous protection involved in cardioprotection after I/R injury [11].

Growing evidence suggests that soluble receptor for advanced glycation end-products (sRAGE) most likely protects the heart [12]. The ligand–receptor for the advanced glycation end-products (RAGE) axis has emerged as a novel pathway involved in a wide spectrum of diseases, including diabetes mellitus, chronic renal failure and I/R [13]. Circulating soluble forms of RAGE (sRAGE), arising from receptor ectodomain shedding and splice variant endogenous secretory RAGE (esRAGE) secretion, may counteract RAGE-mediated pathogenesis, by acting as a decoy [11]. Our previous work [14] showed that endogenous sRAGE was down-regulated by I/R in the rat. Multiple studies have supported evidence that interactions for ligand/RAGE play a central role in myocardial I/R injury [15,16]. The validity of these observations and their clinical relevance remain undefined. If the aforementioned is proven, myocardial ischemia and infarction may represent another potential target for therapeutic modulation. Therefore, sRAGE may be a novel signaling mechanism that can protect against I/R injury. In this study, we aimed to investigate the effect of sRAGE on cardiomyocyte apoptosis and examine multiple downstream apoptotic signals in rat neonatal cardiomyocytes treated with hypoxia/reoxygenation (H/R) *in vitro*.

## 2. Results

### 2.1. sRAGE Prevented the Reduction of Cell Viability Following H/R

**Figure 1:**
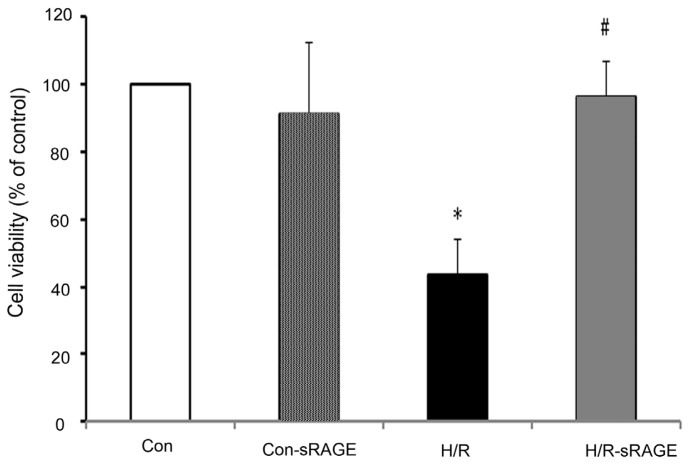
Cell viability of neonatal rat cardiomyocytes. Cell viability was quantified by the MTT assay. Con, no treatment; H/R, 3-h hypoxia followed by 2-h reoxygenation; H/R- sRAGE, sRAGE for 10 min, then 3-h hypoxia before 2-h reoxygenation; Con-sRAGE, sRAGE alone. Dose of sRAGE was 900 ng/mL. Data are the mean ± SD of optical density from the MTT assay. (* *p* < 0.01 compared with the control, # *p* < 0.01 compared with H/R, *n* = 8).

Treatment with H/R reduced the number of viable cells, and sRAGE pre-treatment increased cell viability as determined by the MTT assay. Cell viability in the H/R group accounted for 43.8% of the control group. Compared with the H/R group, sRAGE incubation increased viability from 43.8% ± 10.5% to 96.6% ± 10.1% (*n* = 8, *p* < 0.01). sRAGE alone had no effect on cell viability (Figure 1).

### 2.2. sRAGE Inhibited LDH Leakage Induced by H/R

**Figure 2:**
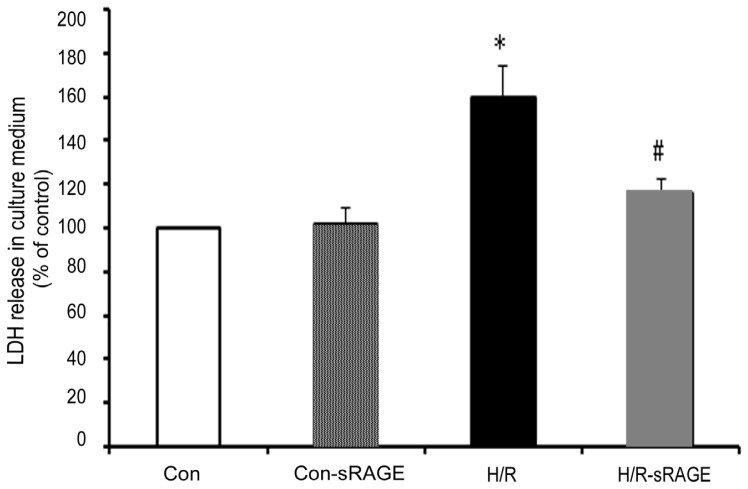
LDH leakage in medium of cardiomyocytes. LDH content in cell-culture medium was quantified. Con, no treatment; H/R, 3-h hypoxia followed by 2-h reoxygenation; H/R -sRAGE, sRAGE for 10 min, then 3-h hypoxia before 2-h reoxygenation; Con-sRAGE, sRAGE alone. Dose of sRAGE was 900 ng/mL. Data is the mean ± SD (* *p* < 0.01 compared with the control, # *p* < 0.01 compared with H/R, *n* = 8).

Treatment with H/R increased the amount of cardiomyocyte LDH leakage to 59.6% in comparison with the control group. sRAGE pre-treatment significantly reduced the amount of cardiomyocyte LDH leakage induced by H/R from 159.6% ± 14.7% to 115.6% ± 6.7% (*n* = 8, *p* < 0.01). sRAGE alone had no effect on cardiomyocyte LDH leakage (Figure 2).

### 2.3. Exogenous Administration of sRAGE Inhibited H/R Induced Cardiomyocyte Apoptosis in vitro

**Figure 3:**
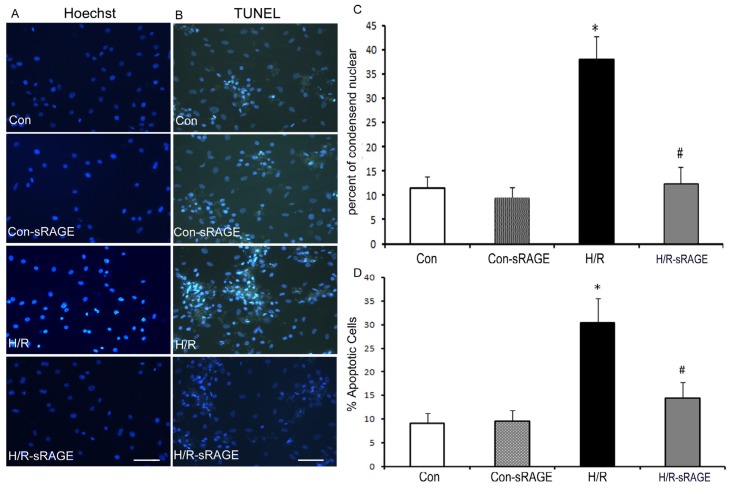
Effect of sRAGE on apoptosis. Effect of sRAGE during H/R on apoptosis by Hoechst 33258 (**A**) and TUNEL staining (**B**). Con, no treatment; Con-sRAGE, sRAGE alone; H/R, 3-h hypoxia followed by 2-h reoxygenation; H/R-sRAGE, sRAGE for 10 min, then 3-h hypoxia before 2-h reoxygenation. The scale bar indicates 50 μM. Apoptotic ratio further analyzed by Hoechst 33258 staining (**C**) and TUNEL staining (**D**). Dose of sRAGE was 900 ng/mL. Data are the mean ± SD (* *p* < 0.01 compared with the control, # *p* < 0.01 compared with H/R).

To determine the effect of sRAGE pretreatment on apoptosis induced by H/R in cultures of neonatal rat cardiac myocytes, we first examined cell morphology by phase-contrast microscopy and nuclear morphology by Hoechst and terminal deoxynucleotidyl transferase-mediated dUTP nick-end labeling (TUNEL) staining. Hoechst 33258 staining revealed that control cell nuclei had regular contours and round or elliptical shapes. In contrast, H/R cells showed smaller nuclei and condensed chromatin. Incubation with sRAGE improved morphological features and decreased the number of apoptotic cells induced by H/R. (Figure 3A). The apoptosis ratio was higher for H/R as compared to the control (27.9% ± 5.9% *vs.* 11.4% ± 2.4%, respectively; *n* = 8, *p* < 0.01). Compared with the H/R treatment, sRAGE pre-treatment decreased H/R-induced apoptosis from 27.9% ± 5.9% to 9.4% ± 0.7% (*n* = 8, *p* < 0.01). sRAGE alone had no effect on cardiomyocyte apoptosis (Figure 3B). The apoptotic results were further confirmed by TUNEL staining (Figure 3C). Almost no TUNEL-positive cells could be found with control treatment, but many TUNEL-positive cells were found with H/R. The number of TUNEL-positive cells was higher for H/R as compared to the control (30.4% ± 5.2% *vs.* 9.2% ± 2.0%, respectively; *n* = 8, *p* < 0.01). Compared with the H/R treatment, sRAGE pre-treatment decreased H/R-induced apoptosis from 30.4% ± 5.2% to 14.5% ± 3.3% (*n* = 8, *p* < 0.01). sRAGE alone had no effect on cardiomyocyte apoptosis (Figure 3D).

### 2.4. sRAGE Inhibited Mitochondrial Depolarization and mPTP Opening Induced by H/R

**Figure 4:**
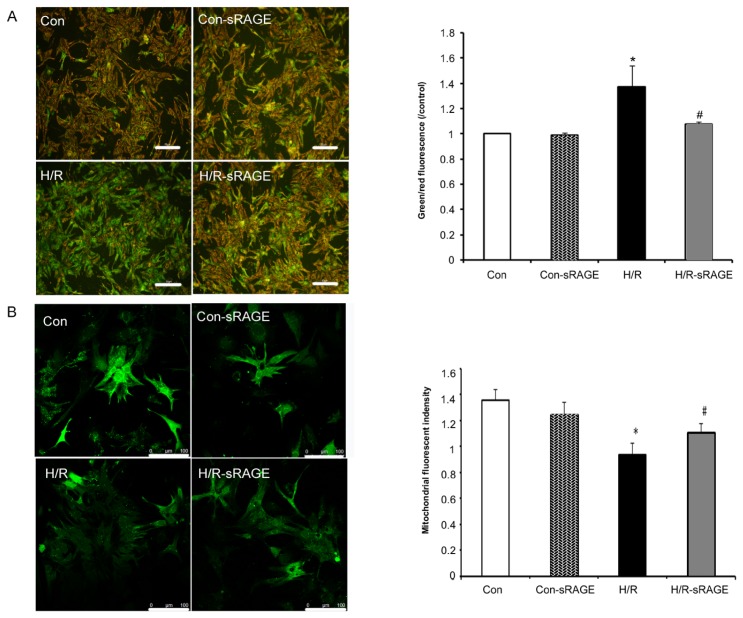
Effect of sRAGE on mitochondrial membrane potential (Δψ_m_) and mitochondrial permeability transition pore (mPTP) opening. To monitor changes in Δψ_m_ (**A**), cells were incubated with 10 μg/mL JC-1 for 20 min at 37 °C. Data is presented as the relative ratio of green to red fluorescence intensity, which indicates ΔΨm depolarization. To reveal mPTP opening (**B**), cells were co-loaded for 15 min with 2 μM calcein-AM and 4 mM CoCl_2_ at 37 °C. The rates of calcein-AM loading and exit were measured by fluorescent signal recording every 5 min by confocal microscopy. Con, no treatment; H/R, 3-h hypoxia followed by 2-h reoxygenation; H/R-sRAGE, sRAGE for 10 min, then 3-h hypoxia before 2-h reoxygenation; Con-sRAGE, sRAGE alone. Dose of sRAGE was 900 ng/mL. Data are the mean ± SD (* *p* < 0.01 *vs.* the control, # *p* < 0.01 *vs.* H/R; *n* = 8). The scale bar indicates 50 μM for Figure 4A and 100 μM for Figure 4B.

Δψ_m_ is an important mediator and monitor of key cellular processes. In addition, it is a highly sensitive indicator of the energetic state of mitochondria and the health of cells [17]. Detection of mitochondrial permeability provided an early indication of the initiation of cellular apoptosis. Using JC-1, H/R increased Δψ_m_ depolarization by 37% (*n* = 8, *p* < 0.01) compared to the controls. Compared with H/R, sRAGE pre-treatment decreased mitochondrial depolarization from 1.37 ± 0.17 to 1.08 ± 0.02 (*n* = 8, *p* < 0.01). sRAGE alone had no effect on Δψ_m_. However, sRAGE changed H/R-induced Δψ_m_ (Figure 4A).

The occurrence and mode of mPTP opening in intact cells was investigated by monitoring the fluorescence of mitochondrial-entrapped calcein with the calcein-AM and CoCl_2_ co-loading method. Compared with the controls, H/R decreased the fluorescence intensity from 1.36 ± 0.08 to 0.94 ± 0.09 (*n* = 8, *p* < 0.01), which indicated increased mPTP opening; compared with H/R, sRAGE pre-treatment inhibited mPTP opening, with a fluorescence increase from 0.94 ± 0.09 to 1.11 ± 0.07 (*n* = 8, *p* < 0.01). sRAGE alone had no effect on mPTP opening. Therefore, exogenous administration sRAGE inhibited H/R-induced mPTP opening (Figure 4B).

### 2.5. sRAGE Inhibited H/R-Induced Mitochondrial Cytochrome c Release and the Increase of Caspase-3 and Caspase-9 Activity during H/R

**Figure 5:**
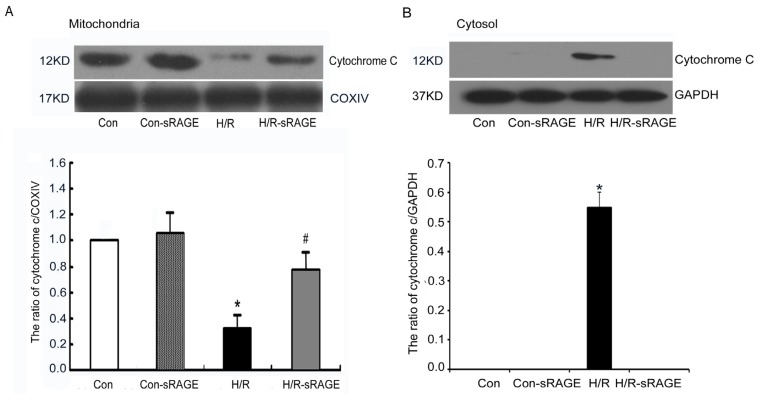
Effect of sRAGE on mitochondrial cytochrome c leakage. (**A**) Western blot analysis of mitochondrial cytochrome c protein (upper panel) and cytochrome oxidase IV (COXIV) (loading control; lower panel). Densitometry of cytochrome c levels normalized to COXIV in each treatment condition. Data are the mean ± SD (* *p* < 0.01 *vs.* control, # *p* < 0.01 *vs.* H/R; *n* = 4). (**B**) Western blot analysis of cytosolic cytochrome c protein (upper panel) and GAPDH (loading control; lower panel). Densitometry of cytochrome c levels normalized to GAPDH in each treatment condition. Dose of sRAGE was 900 ng/mL. Data are the mean ± SD (* *p* < 0.01 *vs.* control, # *p* < 0.01 *vs.* H/R; *n* = 4).

In the mitochondria-mediated apoptotic pathway, disruption of Δψ_m_ and release of cytochrome c activates a cascade of caspases, including caspase-3, a key and irreversible point in the development of apoptosis. Compared with the controls, H/R induced serious damage to the mitochondrial membranes, resulting in the release of cytochrome c from mitochondria. H/R significantly decreased cytochrome c in the mitochondria by 66% (0.34 ± 0.06, *n* = 4, *p* < 0.01). However, cytochrome c was significantly preserved in the H/R-sRAGE group. Compared with the H/R group, cytochrome c in the mitochondria in the H/R-sRAGE group increased from 0.34 ± 0.06 to 0.80 ± 0.08 (*n* = 4, *p* < 0.01). sRAGE alone had no effect on the release of cytochrome c (Figure 5). Few cytochrome c in the cytoplasm were found in the control group, while H/R significantly increased cytochrome c in the cytoplasm (0.55 ± 0.05, *n* = 4, *p* < 0.01). sRAGE pretreatment completely inhibited H/R-induced cytochrome c release into the cytoplasm.

**Figure 6:**
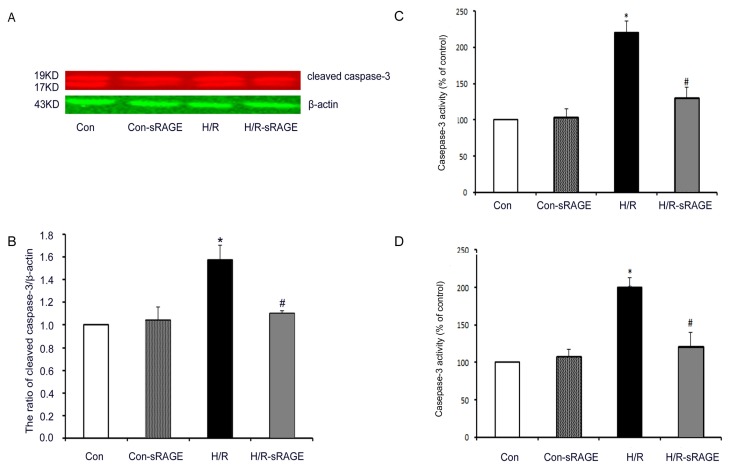
Effect of sRAGE on the level of cleaved caspase-3. (**A**) Western blot analysis of cleaved caspase-3 (upper panel) and β-actin (loading control; lower panel); (**B**) Densitometry of cleaved caspase-3 normalized to β-actin in each treatment condition in (**A**); (**C**) Caspase-3 activity in lysates of cardiomyocytes; (**D**) Caspase-9 activity in lysates of cardiomyocytes. Data in (**C**) and (**D**) were expressed as percentage of control group. Dose of sRAGE was 900 ng/mL. Data are the mean ± SD (* *p* < 0.01 *vs*. control, # *p* < 0.01 *vs.* H/R; *n* = 4).

Compared with the controls, H/R significantly increased the levels of cleaved caspase-3 by 57% (1.57 ± 0.13, *n* = 4, *p* < 0.01). sRAGE significantly attenuated the H/R-induced increase in cleaved caspase-3 from 1.10 ± 0.02 to 1.57 ± 0.13 (*n* = 4, *p* < 0.01). sRAGE alone had no effect on cleaved caspase-3 (Figure 6A,B). Compared with the controls, H/R significantly increased caspase-3 activity by 120% (2.20 ± 0.16, *n* = 4, *p* < 0.01). sRAGE significantly inhibited the H/R-induced increase in caspase-3 activity from 2.20 ± 0.16 to 1.30 ± 0.15 (*n* = 4, *p* < 0.01). sRAGE alone had no effect on caspase-3 activity (Figure 6C). Similar results appeared in caspase-9, compared with the controls, H/R significantly increased caspase-9 activity by 100% (2.00 ± 0.13, *n* = 4, *p* < 0.01). sRAGE significantly reduced the H/R-induced increase in caspase-9 activity from 2.00 ± 0.13 to 1.21 ± 0.19 (*n* = 4, *p* < 0.01). sRAGE alone had no effect on caspase-9 activity (Figure 6D). Therefore, sRAGE inhibits apoptosis by attenuating H/R-induced increase in caspase-3 and caspase-9 activity.

### 2.6. sRAGE Inhibited the Increase of the Ratio of Bax to Bcl-2 following H/R Injury

**Figure 7:**
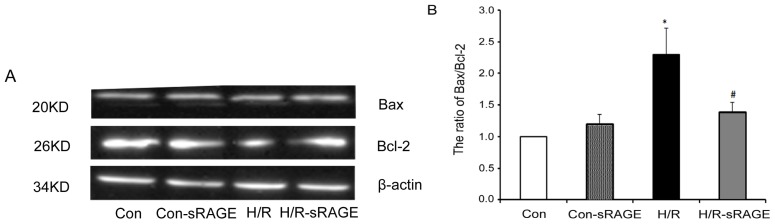
Effect of sRAGE on the ratio of Bax to Bcl-2. (**A**) Western blot analysis of Bax and Bcl-2 (upper panels) and β-actin (loading control; lower panel). (**B**) Densitometry of Bax and Bcl-2 levels normalized to β-actin in each treatment condition in (**A**). Dose of sRAGE was 900 ng/mL. Data are the mean ± SD (* *p* < 0.01 *vs.* control. # *p* < 0.01 *vs.* H/R; *n* = 4).

The Bcl-2 family proteins Bax and Bcl-2 play important roles in initiating the mitochondrial death cascade. Western blot analysis was used to investigate the expression of pro-apoptotic Bax and anti-apoptotic Bcl-2 underlying apoptosis reduced by sRAGE. Compared with the control group, H/R significantly increased the ratio of Bax to Bcl-2 by 130% (2.30 ± 0.41, *n* = 4, *p* < 0.01) (Figure 7). Compared with H/R, sRAGE significantly decreased the H/R-induced increase in ratio of Bax to Bcl-2 from 2.30 ± 0.41 to 1.40 ± 0.14 (*n* = 4, *p* < 0.01). sRAGE alone had no effect on the ratio of Bax to Bcl-2. Thus, sRAGE inhibited apoptosis induced by H/R through increasing the ratio of Bax to Bcl-2.

## 3. Materials and Methods

### 3.1. Materials

Neonatal Wistar rats (1–3 days old) were provided by the Animal Department of Capital Medical University. All experiments were performed in accordance with animal protocols approved by the local authorities in Beijing, China. Fetal calf serum (FCS), Dulbecco’s modified Eagle’s medium (DMEM), and streptomycin/penicillin were from Gibco BRL (Life Technologies, Paisley, UK). Solid-phase extraction (SPE) cartridges (Oasis HLB) were from Waters (Milford, MA, USA). A cytotoxicity detection kit (to measure lactate dehydrogenase [LDH] release) was from Roche (04744934001, CH-4070 Basel, Switzerland). JC-1 and calcein-AM were from Invitrogen (Carlsbad, CA, USA), and CoCl_2_ was from Sigma (St. Louis, MO, USA). Trizol reagent was from Promega (Madison, WI, USA). sRAGE was from Adipo Bioscience Incorporation(Santa Clara, CA, USA). Anti-β-actin, -GADPH antibody were from Santa Cruz Biotechnology (Santa Cruz, CA, USA). Anti-cytochrome c, -COXIV, -Bax, -BCL-2, and -cleaved caspase-3 antibodies were from Cell Signaling Technology (Danvers, MA, USA). Caspase-9 and caspase-3 activity assay kit were provided by Beyotime Institute of Biotechnology (Nanjing, China). Other chemicals and reagents used were of analytical grade.

### 3.2. Isolation and Culture of Neonatal Rat Cardiomyocytes

The heart of rats were excised, and the ventricular myocardium was cut into small pieces (~2 mm^3^) in phosphate-buffered saline (PBS) buffered with trypsin (1.125 mg/mL), collagenase I (1 mg/mL) and collagenase II (0.5 mg/mL). Tissue was incubated on a shaker for 20 min at 37 °C with agitation at 100 rpm. Tissue pieces were allowed to settle, and the supernatant containing myocytes was collected, suspended, and centrifuged at 1000 rpm for 10 min. The cell pellet was re-suspended and stored at 37 °C. Cells were then re-suspended in DMEM with 20% (*v*/*v*) FCS and 0.5% (*v*/*v*) penicillin/streptomycin for 30 min to facilitate separation of ventricular myocytes from the faster-attaching non-myocytes. Ventricular myocytes were then collected and plated on gelatin-coated dishes. Cells were used for experiments after demonstrating rhythmic contractions (48–72 h).

### 3.3. Study Groups and Experimental Protocol

Cultured neonatal rat cardiomyocytes were randomly divided into the following groups for treatment: Control group (Con): cardiomyocytes were continuously cultured for 5 h without any treatment in normal culture medium; H/R group: cells were subjected to 3 h simulated hypoxia (approximately 2% O_2_/5% CO_2_), then 2 h of normoxia (approximately 20% O_2_/5% CO_2_) as described [18]; H/R-sRAGE group: 900 ng/mL sRAGE added to the culture medium 10 min before hypoxia [19,20]; Con-sRAGE group, 900 ng/mL sRAGE added to the culture medium alone without hypoxia. For controls, equivalent volumes of medium were added. Only cultures that consisted of >95% rhythmically beating cells, determined by counting 300 cells in 3 different fields, were used in the analysis.

### 3.4. Cell Viability Assay

Cell viability was determined using the 3-(4,5)-dimethylthiazol (-z-yl)-3,5-di-phenytetrazoliumromide (MTT) assay [21]. Cells were cultured in 96-well plates, and MTT was added to each well under sterile conditions (with a final concentration of 5 mg/mL) immediately after 2 h of reoxygenation. Plates were then incubated for 4 h at 37 °C. The supernatant was removed, and dimethylsulfoxide was added. Plates were then agitated on a plate shaker. The absorbance of each well was measured at 490 nm with a Wellscan MK 3 automated EIA Analyzer (Labsystems Dragon, Taiwan). Viability of control cells was considered to be 100%, and that of other cells was expressed as a percentage of the control.

### 3.5. Assay of LDH Activity

LDH kit was used to measure the activity of LDH released into the medium using by an automatic biochemistry analyzer (Roche, 04744934001, CH-4070 Basel, Switzerland) according to the manufacturer’s instructions. Briefly, 50 mL of medium and 50 mL of a mix of reagent A and B were co-incubated for 30 min. The absorbance was detected at 492 nm with the use of a spectrophotometer (Labsystems Dragon, Taiwan). Results were demonstrated with 100% in the control group, and that of other cells was expressed as a percentage of the control.

### 3.6. Apoptosis Assessment

Apoptosis of cardiomyocytes was determined by nuclear staining with the chromatin dye Hoechst 33258 and TUNEL as reported previously [3,22]. Cells were fixed with 10% (*v*/*v*) paraformaldehyde for 15 min at 4 °C. After three washes in PBS, cells were permeabilized with 0.2% (*v*/*v*) Triton X-100 for 10 min at room temperature, then incubated with 50 μL TUNEL reaction mixture (Promega, San Luis Obispo, CA, USA) for 30 min at 37 °C. Diaminobenzidine was used to generate an insoluble colored substrate at the site of DNA fragmentation. Cells were finally exposed to Hoechst 33258 (10 μg/mL) (Sigma, St. Louis, MO, USA) for 5 min and examined under a microscope (OLYMPUS BX63, Japan). Apoptotic cells were identified on the basis of distinctively condensed or fragmented nuclear morphology, and apoptotic cell counts were expressed as a percentage of the total number of nuclei counted.

### 3.7. Measurement of MMP by Florescent JC1

MMP was estimated by monitoring fluorescence aggregates of JC-1 (tetrachloro-1,1′,3,3′-tetraethyl- 6′,6,5′,5-benzamidazolocarbocyanin iodide), which demonstrated MMP function. The formation of JC-1 aggregates and their fluorescence linearly correspond to increases in membrane potential [23]. Regions of high mitochondrial polarization are indicated by red fluorescence due to J-aggregate formation by the concentrated dye. Depolarized regions are indicated by the green fluorescence of the JC-1 monomers. After treatment, cells plated on 24-well plates were incubated with 10 μg/mL JC-1 for 20 min at 37 °C in a humidified incubator, then washed twice with PBS for detection of the fluorescent ratio (for red fluorescence: excitation, 490 nm and emission, 590 nm; for green fluorescence: excitation, 490 nm and emission, 527 nm). Fluorescent intensity was observed using a microscope (Olympus IX71; Japan). Regions of interests were randomly selected and zoomed in the same frames. Data are presented as the relative ratio of green to red fluorescence intensity, which indicates mitochondrial membrane potential depolarization.

### 3.8. Determination of mPTP Opening

Transient mPTP opening was directly assessed by co-loading cells with calcein-AM and CoCl_2_ as previously described [24]. Calcein-AM was permeable to intact membranes but CoCl_2_ was not permeable to intact mitochondrial membranes. Thus, the conditions allowed for monitoring of calcein fluorescence in mitochondria of intact cells. Inner mitochondrial membrane permeability to the fluorescent dye calcein-AM indicated opening of the mPTP in intact cells. Calcein fluorescence in the cytosol was quenched by the addition of CoCl_2_, and calcein fluorescence intensity in mitochondria was stronger when mPTP was closed than when open. In brief, cardiomyocytes were loaded for 15 min with 2 μM calcein-AM at room temperature and then washed free of calcein-AM and CoCl_2_. Rates of calcein-AM loading and exit were measured by recording the fluorescent signal every 5 min with the Turner Quantech Digital Filter Fluorometer (Barnstead/Thermolyne, USA; excitation filter NB488 and emission filter SC515) and calculated as the percent change to maximal fluorescent signal.

### 3.9. Isolation of Mitochondria

Isolation of mitochondria was performed according to instructions from the Mitochondria/Cytosol Isolation Kit for Cultured Cells. Cells were harvested and homogenized in 1 mL of ice-cold Mito-Cyto Buffer with a Dounce homogenizer. After two centrifugations at 800*g* for 5 min each at 4 °C, the supernatant was collected, transferred to a fresh microcentrifuge tube, and centrifuged at 12,000*g* for 10 min at 4 °C. The pellet, which contained the mitochondria, was re-suspended in 30 μL of Mito-Cyto Buffer for further use.

### 3.10. Western Blot Analysis

Total protein (80 or 100 μg) isolated from neonatal rat cardiomyocytes was separated by SDS-PAGE and blotted onto polyvinylidene difluoride (Hybond ECL, Amersham, USA). Membranes were blocked with 5% (*v*/*v*) nonfat dry milk in Tris-buffered saline-Tween (TBST; 10 mM Tris, 150 mM NaCl, and 0.1% (*v*/*v*) Tween-20) for 2 h at room temperature before being incubated overnight at 4 °C with anti-Bcl-2 mouse polyclonal antibody (1:1000), anti-Bax rabbit polyclonal antibody (1:1000), anti-cytochrome c rabbit polyclonal antibody (1:1000), anti-caspase-3 rabbit polyclonal antibody (1:1000), anti-β-actin mouse monoclonal antibody (1:1000) and anti-COXIV monoclonal antibody (1:1000 in 0.5% (*v*/*v*) milk in TBST). After three washes with TBST, membranes were incubated with horseradish peroxidase-labeled or fluorescent-labeled secondary antibody or in TBST. After extensive washing in TBST, membranes were developed using an enhanced chemiluminescence kit.

### 3.11. Caspase Activity Assay

Caspase activities were determined by a colorimetric assay based on the ability of caspase-3 and caspase-9 to change acetyl-Asp-Glu-Val-Asp p-nitroanilide (Ac-DEVD-pNA) and acetyl-Leu-Glu-His-Asp p-nitroanilide (Ac-LEHD-pNA) into a yellow formazan product (*p*-nitroaniline (pNA)), respectively. An increase in absorbance at 405 nm was used to quantify the activation of caspases activities. Cardiomyocytes were collected and rinsed with cold PBS, then lysed by lysis buffer for 15 min on ice. Cell lysates were centrifuged at 20,000× *g* for 10 min at 4 °C. Caspase-3, -9 activities in the supernatant were assayed using the kit. The caspase activities were expressed as percentage of enzyme activity compared to control. All the experiments were carried out in triplicates.

### 3.12. Statistical Analysis

All values are expressed as the mean ± SD and were analyzed using SPSS v.11.5 (version 11.5; SPSS Inc., Chicago, IL, USA, 2003). Differences between groups were assessed by one-way ANOVA and LSD test. A *p*-value of less than 0.05 was considered statistically significant.

## 4. Discussion

sRAGE is the soluble isoform of RAGE that exists in the circulation. RAGE is a 35 kD cell-bound transmembrane receptor of the immunoglobulin superfamily [25]. RAGE consists of 3 immunoglobulin-like extracellular regions (1 V-type and 2 C-type domains), a short transmembrane region, and a cytoplasmic tail [26]. RAGE interacts with several pro-inflammatory molecules, including advanced glycation endproducts (AGEs), S100 proteins/calgranulins [27], amphoterin or high-mobility group box 1 protein (HMGB1) [28], amyloid-β peptide [29], transthyretin [30] and Mac-1 [31]. Following activation of RAGE, these pro-inflammatory molecules may synergistically induce a pathophysiological response on different cell types implicated in these disease processes, including endothelial cells, vascular smooth muscle cells, monocytes/macrophages, lymphocytes, cardiomyocytes, neurons and podocytes [32,33]. A fundamental consequence of RAGE signaling is the enhanced generation of reactive oxygen species, critically involved in the activation of the transcription factor NF-κB and in the mechanisms of endothelial dysfunction, platelet activation and amplification of the inflammatory response [34]. Under physiological or pathophysiological conditions, cells can release sRAGE into the extracellular space.

sRAGE is a product of both cleavage of the extracellular region of RAGE and the endogenous secreted form of RAGE (esRAGE) [35–37]. While the biological function of sRAGE has not been clearly defined, one proposed pathological role is that it works as a competitive inhibitor of ligand-RAGE interaction and subsequent downstream signaling. Furthermore, sRAGE may also serve as a scavenger receptor for circulating AGEs and other RAGE ligands. The biological importance of sRAGE within the ligand-RAGE axis is only recently beginning to be understood; however, few studies have focused on the effect of sRAGE following I/R (ischemia/reperfusion).

To gain mechanistic insight into the cardioprotective effects of sRAGE in H/R, we used a cultured neonatal cardiomyocyte model of H/R. Consistent with previous investigations [38], the use of two complementary techniques to assess cell death after H/R revealed reduced cell viability and increased LDH leakage. To demonstrate that sRAGE is an endogenous protective pathway, we administered sRAGE to H/R cardiomyocytes. Our results showed that sRAGE, when added before hypoxia, significantly decreased cellular LDH leakage and increased cell viability in neonatal rat cardiomyocytes treated with H/R. The study suggested that sRAGE can protected against myocardium injury following H/R, which is consistent with other studies [39]. Falcone *et al.* [40] performed the first clinical study to investigate sRAGE levels in humans. They showed significantly lower sRAGE levels in patients with angiographically proven coronary artery disease (CAD) than controlled subjects. However, the relationship between sRAGE and CAD is unclear. Bucciarelli *et al.* [11] explored the role of RAGE in hearts subjected to I/R injury using an isolated perfused heart model. Compared to vehicle treated rats, treatment with sRAGE reduced ischemic injury and improved functional recovery of the myocardium. Experiments revealed that hearts from homozygous RAGE null mice were significantly protected from the adverse impact of I/R compared to wild-type mouse hearts, as indicated by decreased release of LDH (a measure of necrosis), improved functional recovery and increased levels of ATP. Interestingly, data revealed that I/R itself produced the pre-AGE MGO, thus providing a novel mechanism for I/R, which even in the absence of diabetes, recruits and activates RAGE [11]. RAGE is critically implicated in cardiac cell death after I/R injury. Alexey Aleshin *et al*. [15] performed a study to investigate the key role for RAGE in the pathogenesis of myocardial injury induced by transient occlusion and reperfusion of the LAD. Findings identified RAGE as a major upstream regulator of the I/R injury-provoking signaling pathways in the heart. Exogenous sRAGE may attenuate myocardium injury after I/R in rat models. All of these data strongly suggest that sRAGE was involved in cardioprotection, but the protective mechanism by which endogenous and exogenous sRAGEs initiate their action are unclear. In this study, sRAGE was exogenously administered to caridomyocytes. The concentration of sRAGE used in the study was determined by our pilot study. We also referenced the dose from other studies [19,20].

Cardiomyocyte apoptosis is one of the major contributors of myocardial injury after I/R. Blocking the apoptosis process could prevent the loss of contractile cells and minimize cardiac injury induced by I/R [41,42]. Apoptosis plays an important role in I/R injury pathogenesis. We aimed to investigate the effect of sRAGE on neonatal rat cardiomyocytes to explore whether sRAGE is involved in cardioprotection in H/R by inhibiting apoptosis. Neonatal rat cardiomyocytes were exposed to H/R alone or treated with sRAGE before H/R. Results revealed that H/R accelerated apoptosis in our cell-culture model. As with H/R alone, sRAGE attenuated cardiomyocyte apoptosis from 27.9% ± 5.9% to 9.4% ± 0.7% (*p* < 0.05). sRAGE significantly protected neonatal rat cardiomyocytes against H/R-induced apoptosis as evaluated by Hoechst staining analysis. Therefore, sRAGE is an important regulator of neonatal rat cardiomyocyte apoptosis.

The high-energy demands of cardiac function are supplied by ATP and produced mainly by mitochondria through oxidative phosphorylation, glycolysis and the Krebs cycle [43,44]. Ischemic events can greatly alter mitochondrial function and thereby decrease cardiac efficiency. This damage ultimately contributes to contractile dysfunction, mPTP opening, and cell death. mPTP formation may be the event that leads to irreversible changes in cellular function and cell death [45]. During the early minutes of reperfusion, postconditioning reduces oxidative stress and inhibits mPTP opening, independent of altered oxidative phosphorylation or Δψ_m_ [46]. We continued to explore whether sRAGE protects the myocardium using the mitochondrial apoptosis method. Our results demonstrated that H/R induced Δψ_m_ depolarization levels and mPTP opening. sRAGE significantly inhibited Δψ_m_ depolarization and inhibited mPTP opening as evaluated by JC-1 staining and calcein-AM and CoCl_2_ co-loading analysis. Thus, sRAGE inhibited apoptosis by decreasing Δψ_m_ and mPTP opening.

A diverse array of intrinsic and extrinsic stimuli regulates endothelial cell apoptosis by modulating the balance between the pro-apoptotic caspases and various anti-apoptotic proteins such as Bcl-2. Our results showed that sRAGE significantly inhibited mitochondrial cytochrome c release and caspase-3 and caspase-9 activity following H/R as well as decreased the ratio of Bax to Bcl-2. The effect of sRAGE on caspase-3 in cardiomyocytes was consistent with another study in cardiac microvascular endothelial cells. Yi Liu *et al.* [19] reported sRAGE attenuated I/R induced LDH release and caspase-3 activity in cardiac microvascular endothelial cells. Thus, mitochondrial cytochrome c release, caspase-3 and caspase-9 and apoptotic proteins such as Bcl-2 and Bax are involved in the cardioprotection of sRAGE in H/R. As a result, sRAGE induced cardioprotection by inhibiting apoptosis via a caspase-dependent pathway. This apoptosis-suppressive effect of sRAGE may have important clinical implications during H/R.

## 5. Conclusions

Our study revealed that sRAGE induced cardioprotection by inhibiting apoptosis via the mitochondrial pathway in H/R. This cardioprotective effect appeared to involve decreased mPTP opening, attenuation of H/R-mediated mitochondrial cytochrome c release, and reduction of caspase-3 and caspase-9 activity and the ratio of Bax to Bcl-2. Increased sRAGE biosynthesis may contribute to the survival of neonatal rat cardiomyocytes. If confirmed further, the apoptosis-suppressive effect of sRAGE may have important clinical implications during H/R. Modulation of sRAGE biosynthesis may also represent a novel therapeutic strategy for the clinical treatment of H/R.
